# Degradation Physics of High Power LEDs in Outdoor Environment and the Role of Phosphor in the degradation process

**DOI:** 10.1038/srep24052

**Published:** 2016-04-07

**Authors:** Preetpal Singh, Cher Ming Tan

**Affiliations:** 1 Department of Electronics Engineering, Chang Gung University, Wenhua 1st Rd., Guishan Dist., Taoyuan City 33302, Taiwan; 2 Centre for Reliability Science and Technology, Chang Gung University, Wenhua 1st Road, Guishan Dist., Taoyuan City 33302, Taiwan; 3 Department of Mechanical Engineering, Ming Chi University of Technology, 84 Gungjuan Rd., Taishan Dist., New Taipei City 24301, Taiwan; 4 Department of Urology, Chang Gung Memorial Hospital, Taiwan.; 5 Institute of Radiation Research, College of Medicine, Chang Gung University, Taiwan.

## Abstract

A moisture- electrical – temperature (MET) test is proposed to evaluate the outdoor reliability of high power blue LEDs, with and without phosphor, and to understand the degradation physics of LEDs under the environment of combined humidity, temperature and electrical stresses. The blue LEDs with phosphor will be the high power white LEDs. Scanning acoustic microscopy is used to examine the resulted delamination during this test for the LEDs. The degradation mechanisms of blue LEDs (LEDs without phosphor) and white LEDs (LEDs with phosphor) are found to be different, under both the power on (i.e. with 350 mA through each LED) and power off (i.e. without current supply) conditions. Difference in the coefficient of thermal expansion between the molding part and the lens material as well as the heat generated by the phosphor layer are found to account for the major differences in the degradation mechanisms observed. The findings indicate that the proposed MET test is necessary for the LED industry in evaluating the reliability of LEDs under practical outdoor usage environment.

LEDs are considered to be the next generation light source due to its several advantages, including its long life^1^,^2^,^3^ Thus, for practical applications, long life LEDs must be ensured, and many high temperature stress tests are indeed performed by manufacturers for this purpose, with international standards such as TM-21 and LM-80^1^ formulated. As high power LEDs are increasingly employed in outdoor environment as well as some other harsher environment than indoor applications, effect of moisture on their reliability cannot be overlooked as its effect can indeed be important as shown by the literatures^4^,^5^,^6^,^7^,^8^.

In actual outdoor applications, LEDs are subjected to electrical, thermal and humidity stresses simultaneously, but reliability test that consider all these three stresses is rarely found. In this work, the concurrent effect of these three stresses on the reliability of LEDs is investigated, and a test designed for this purpose is proposed, with exploration of the degradation physics under the combined three stresses. This test is called MET to signify moisture-electrical-thermal test. The degradation mechanisms of LEDs under this MET test are compared to that under the conventional single or double stresses as reported in the literatures. For comprehensiveness, we also studied the effect of phosphor layer on the reliability of high power LEDs under this triple stresses by comparing the degradations of blue LED (i.e. LED without phosphor layer) and white LEDs (i.e. blue LEDs with phosphor), so that the role of phosphor for the degradation process under the combined three stresses can be known.

## Experimentation

Two sets of high power OSRAM golden dragon LEDs are chosen, each consists of 40 units of white high power LEDs and 40 blue high power LEDs respectively. Each set is further divided into 2 sub-sets as shown in Table 1 with 20 LEDs in each sub-set. Sub-sets A1 and A2 consists of white and blue LEDs respectively with power on condition and 350 mA constant current is passing through them individually, according to the manufacturing specification. Sub-sets B1 and B2 consists of white and blue LEDs respectively with power-off condition. All sets are placed in a temperature- humidity chamber (*μ* series from Isuzu) for accelerated life test with test condition set as 85%RH/85 °C. These tests are to mimic the practical situation of outdoor environment in humid countries, aerospace and marine environment in an accelerated manner. For example, in marine environment, the RH is high and the high temperature is an accelerated factor. In street lamp applications in humid countries, the RH is high, and thus the high temperature is again an accelerated factor. Power On condition represents the condition where the street lamp is turned on in the evening, and power OFF condition represents the morning condition where street lamp is off.

The electrical measurements for all the LEDs are done using Keithley source meter model 2651A. Their optical measurements are done with system comprises of a 1-m-diameter integrating sphere model SLM-40TS-110902 and a spectro-radiometer model Ocean Optics QE6500. Initial set of measurements are done on all of them to serve as reference baseline for each test sample.

The samples are taken out of the humidity- temperature chamber every 24 hours for their optical and electrical measurements. The setting for all measurements is according to Tan *et al*.^9^ so as to prevent self-heating during measurement. All the measurement is done within 3 hours from the time they are taken out from the test chamber in order to keep the moisture out-diffusion from the package to its lowest. Optical microscope is used to examine any possible visual defects of the LED’s packages after the tests, and scanning acoustic microscope is employed to observe the possible delamination inside the packages as a result of MET tests. The electrical characteristics of these tested LEDs under different conditions are also compared as will be discussed in Results and Discussion Section.

## Results and Discussion

**Figure 1:**
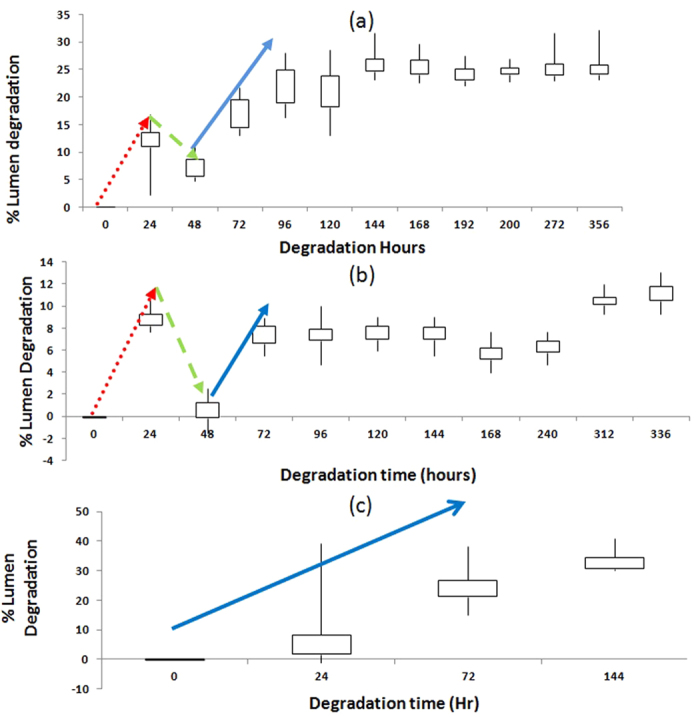
Percentage lumen degradation vs test time for blue and white LEDs tested under different operating conditions. (**a**) Blue LEDs under power-on condition; (**b**) Blue LEDs under power-off condition; and (**c**) White LEDs under power-on condition. Red line (dotted line) denotes the initial degradation; green line (dash line) indicates the lumen recovery and the blue line (solid line) indicates the final degradation for the LEDs.

The percentages lumen degradation vs time for blue LEDs tested under power ON and OFF conditions and white LEDs tested under power ON condition only are shown in Fig. 1. White LEDs under power OFF did not experience any significant degradation during the test.

The lumen degradation time can be divided into 3 stages as observed in Fig. 1. First stage is the initial degradation stage where moisture entering into silicone encapsulation is considered to be the main cause of lumen degradation^4^. The second stage is the recovery stage in which the percentage lumen degradation falls rapidly after the initial degradation. The cause for this lumen recovery will be investigated in the second subsection. The third stage is the final permanent degradation stage due to the prolonged testing. The durations for each of the 3 stages for the blue and white LEDs under different testing conditions are tabulated in Table 2.

Blue LEDs under ON condition degrade up to 12 percent (in the first 24 hours) in the initial degradation stage whereas they degrade only to 7.5 percent (in the first 24 hours) when they are in the OFF condition. Blue LEDs show percentage lumen degradation recovery after the first 24 hours under both the ON and OFF conditions. In the ON condition, blue LEDs show recovery down to 7.5 percent lumen degradation whereas in OFF condition, they can recovery down to 1 percent in next 24 hours of testing. After the recovery stage, LEDs start degrading again. In this stage, blue LEDs under the ON condition show 30 percent lumen degradation after 356 hours whilst they are to 12 percent in the case of the OFF condition in 336 hours.

White LEDs under the ON condition do not show recovery stage but degrading permanently and reach 33 percent lumen degradation in 144 hours, and hence we stop the test for the white LEDs after 144 hours of testing according to the ASISST standard^10^ which sets the largest acceptable lumen degradation at 30%. This shows the negative effect of phosphor in white LEDs when compared to their counterpart blue LEDs under outdoor environment. Extra heat accumulation due to phosphor in white LEDs is believed to be the reason for their higher percentage lumen degradation than blue LEDs as the difference between the blue and white LEDs is only the presence of phosphor, and it has been shown that this phosphor can produce extra heat during the conversion^11^. Indeed, several efforts are made recently to improve the thermal effect of phosphor used in LEDs, such as the placement of phosphor layer etc^12^.

**Figure 2:**
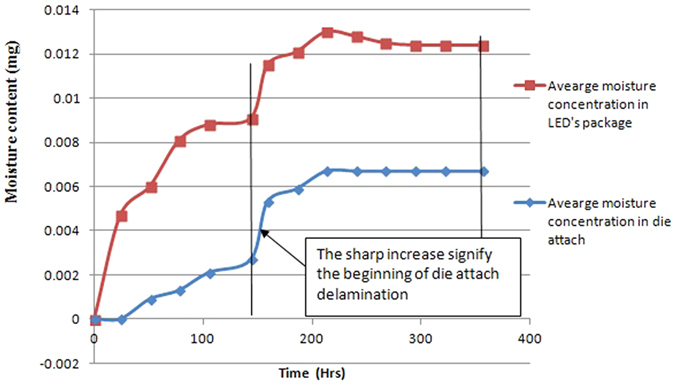
Amount of moisture peneteration into LED package and die attach during testing after the test under OFF condition.

On the other hand, under power OFF condition, white LEDs experience almost zero percentage lumen degradation while blue LEDs experience 12 percent lumen degradation after 336 hours of testing as shown in Table 2. The difference in the degradation observed is due to the much shorter test time for the white LEDs. Finite element analysis of the moisture diffusion into the LED packages as done previously^6^ can provide a much clearer explanation as shown in Fig. 2.

In the figure, we can see that moisture is being absorbed into the encapsulant at the beginning. At around 132 hours, the moisture content in the encapsulant is saturated, and moisture begin to enter into the die attach material, and before 132 hours, the amount of moisture in the die attach material is very low. As moisture continues to enter into the die attach material, delamination at the die attach will occur, and this increase the thermal resistance of the dice, and reduce the lumen output significantly as have been reported by Tan and Singh^7^. The die attach delamination begin around 150 hours as seen in Fig. 2. Therefore, very little lumen degradation is observed for the white LEDs as die attach material is still good, and the heat generated from the phosphor during lumen testing can also help to evaporate some moisture in the encapsulant. As a result, no significant lumen degradation is observed. On the other hand, for the blue LEDs tested for 336 hours, die attach delamination has already occurred, and that the moisture in the die attach cannot be evaporated during testing^6^, this renders significant lumen degradation as observed. The total amount of moisture in the blue LEDs is 40% more than the white LEDs, and this 40% is all in the die attach material.

**Figure 3:**
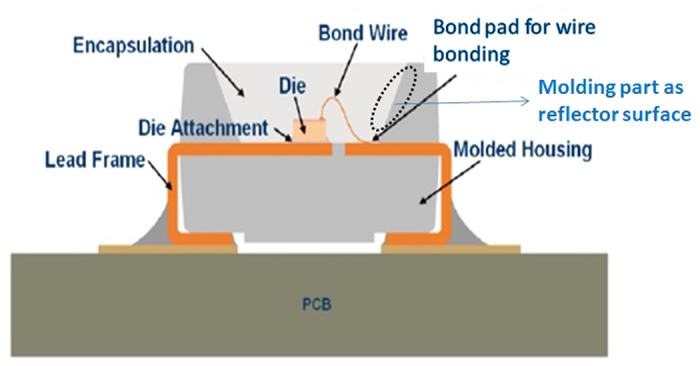
Materials used in LEDs and their possible degradation under MET test^13^.

From Table 2, it is obvious that the LEDs under power-on condition degrade at a faster rate than the one under power-off condition as expected. To understand the mechanisms underlying the increase in percentage lumen degradation as shown in Fig. 1, and to examine the effect of the phosphor presence in white LEDs, we look closer to the structure of LEDs under test as shown in Fig. 3.

For the mechanical protection, LEDs consist of molding part (represented as molded housing in Fig. 3) which holds the entire LED package including LED chip (also known as LED die), encapsulation, lead frame and heat sink. The heat sink is not visible in Fig. 3 as it is embedded inside the molding part under the LED chip and is attached to LED chip using die attachment (also known as die attach as shown in Fig. 3). This molding part is made up of hard silicone whereas the encapsulation of LED chip is silicone rubber as shown in Fig. 3^13^. This molding part functions as a reflector source to enhance the overall LED light output while providing protection to inner LED chip from environmental and mechanical stresses. The silicone encapsulation functions as a lens to direct the light beam as well as provide protection to LED chip from external stress such as dust and moisture.

**Figure 4:**
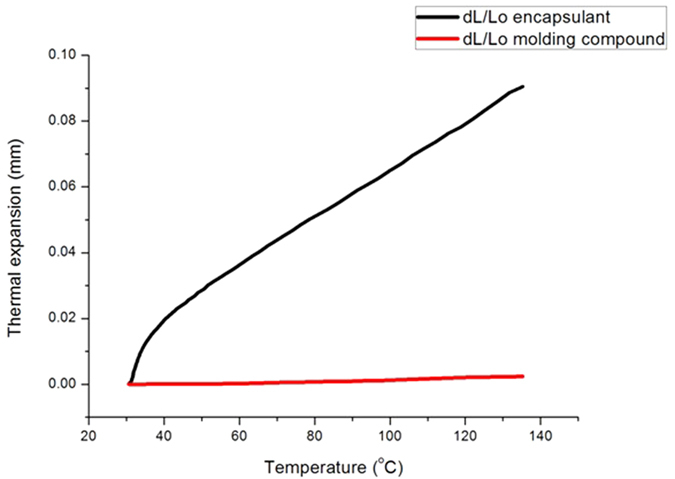
Thermal expansivities of molding part and encapsulant measured at different temperature using NETZSCH dilatometer DIL 402 PC.

According to OSRAM datasheet, poly dimethylsiloxane and phenylsiloxanes are mixed in different concentrations to convert silicone into different forms^13^. Due to the difference in concentration of materials for the molding part and encapsulation silicone, they have different coefficient of thermal expansion^14^,^15^. The Thermal expansivities of molding part and encapsulant are measured using NETZSCH dilatometer DIL 402 PC as shown in Fig. 4.

During the test, chamber temperature reaches 85 °C and as the LEDs are turned ON, LEDs internal temperature can reach 135 °C as mentioned in OSRAM datasheet^16^. With this high temperature during testing, LED molding part and encapsulation rubber undergo expansion, and encapsulant experience 97.5% more expansion than molding part at a temperature of 135 °C.

**Figure 5:**
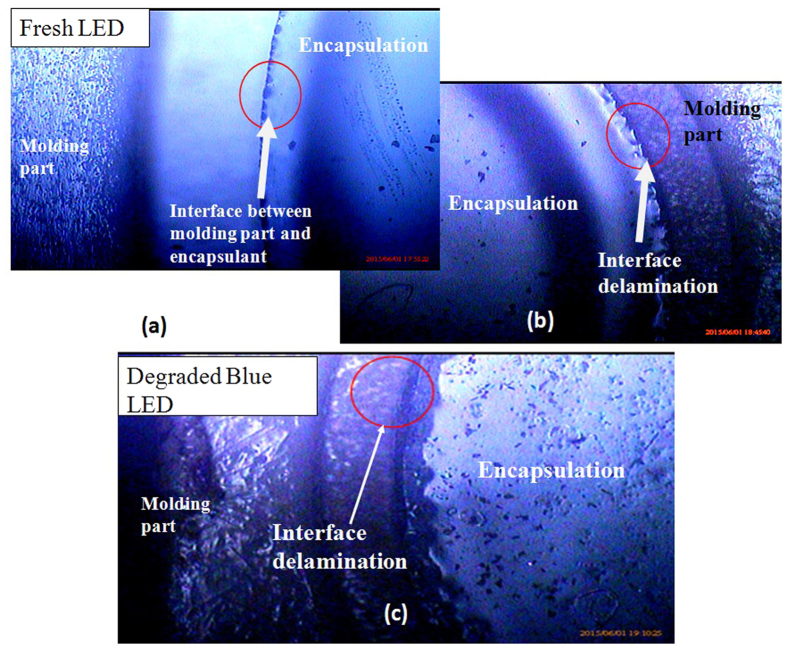
Optical microscope images showing the delamination induced due to difference in thermal expansion at 5× magnification in (**b**) White LEDs and (**c**) Blue LED when compared with (**a**) Fresh LED.

The different in the thermal expansivities of molding part and encapsulant renders differential thermo-mechanical stress generates between the two materials, which lead to delamination, i.e. voids or crack at the interface between molding part and encapsulation. These cracks are found to be visible under the high resolution optical microscope as shown in Fig. 5(a,b) respectively for blue and white LEDs. These cracks and delamination are then the moisture diffusion path in LEDs, causes lumen degradation.

**Figure 6:**
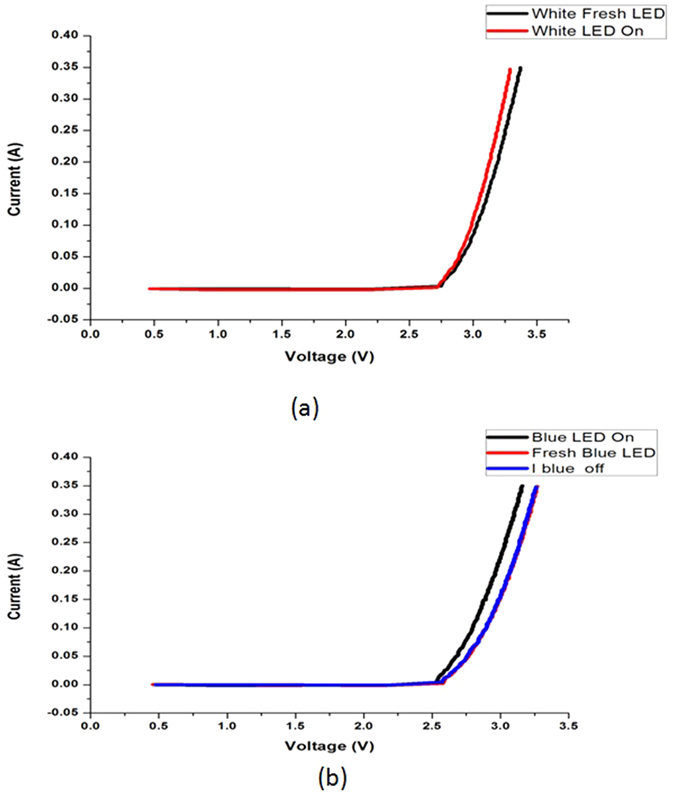
I-V curves for (**a**) White and (**b**) Blue LEDs under different testing conditions.

To further confirm that the delamination observed is indeed a cause of degradation, one sample from each set with maximum reduction in forward voltage (V_f_) is chosen for C-SAM examination. Figure 6 shows typical I-V curves of the white and blue LEDs tested under different conditions. Maximum reduction in forward voltage is chosen as selection criteria because at a constant current, the reduction of LED’s forward voltage indicates a degradation in the thermal resistance of the LED chip due to die attached delamination^7^.

As recovery of degradation is observed during the MET tests (as shown in Fig. 1), two additional units of the blue LEDs under the ON and OFF conditions respectively are chosen to observe the effect of MET test before recovery. Same samples are also chosen to check their possible delamination during test using C-SAM. The test conditions undergone by these six samples and the rationale of their choices are shown in Table 3.

**Figure 7:**
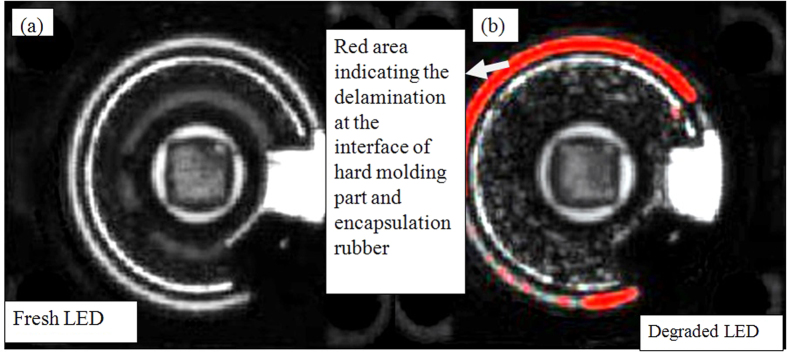
C-SAM images showing the delamination at interface between silicone and molding part of WHITE LEDs. The red area show where the cracks occur when the samples are exposed to high temperature exposures. The left side image shows the fresh LED’s C-SAM image and right side image is the degraded LED’s C-SAM image (sample 1 in Table 3). The die attach area can be identified easily by a square exactly at the center of the LED package.

Scanning acoustic microscopy (SAM) is employed to verify the delamination at the interface of molding part and encapsulation. SAM is a non-destructive microscopy which employs ultra-high frequency sound wave to capture cracks, delamination and voids images present in a device^17^. The C-SAM mode is used to detect the voids and delamination in LED package for the LEDs chosen. The LED die attach area can be identified easily by a square at the center of the LED package in Fig. 7.

In the case of white LEDs under power ON condition, C-SAM images clearly show the delamination induced at the interface between the silicone and molding part as represented by the red areas in Fig. 7(b). The red areas show where the cracks/delamination occur when the samples are exposed to high temperature exposures. On the other hand, there is no delamination for the LEDs under OFF condition or the fresh LEDs as seen in Fig. 7(a), explaining the absence of degradation for the white LEDs under OFF condition. This shows that the delamination is the cause of lumen degradation for the white LEDs under power ON condition, and that it is the internal heat generation that result in this delamination.

**Figure 8:**
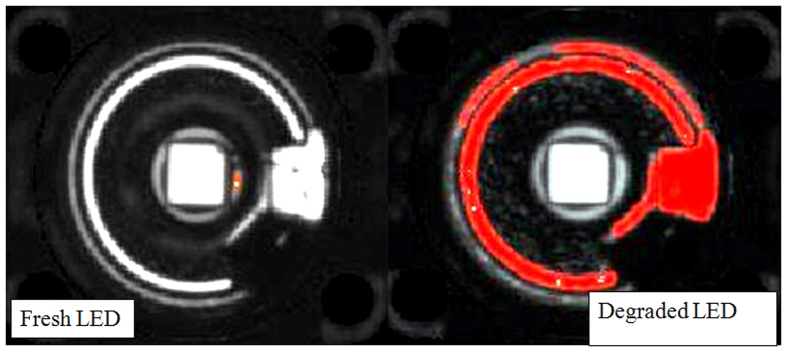
C-SAM images showing the delamination at interface between silicone and molding part of degraded BLUE LED. The red area show where the cracks/delamination occur when the samples are exposed to high temperature exposures. The left side image shows the fresh LED’s C-SAM image and right side image is the degraded LED’s C-SAM image (sample 3 in Table 3). The die attach area can be identified easily by a square exactly at the center of the LED package.

In the case of blue LEDs under ON condition, the delamination is similar to the case of white LEDs under ON condition as clearly observed in Fig. 8. Comparing Figs 7 and 8, one can see that the delamination in the blue LED is more significant than the white LEDs as the red area is larger. This is due to the longer testing time of blue LEDs which is 356 hours as compared to white LEDs which is only 144 hours. On the other hand, the condition of blue LEDs under OFF condition shows no visible delamination, indicating again that it is the internal heat generated from the chip that causes the delamination.

**Figure 9:**
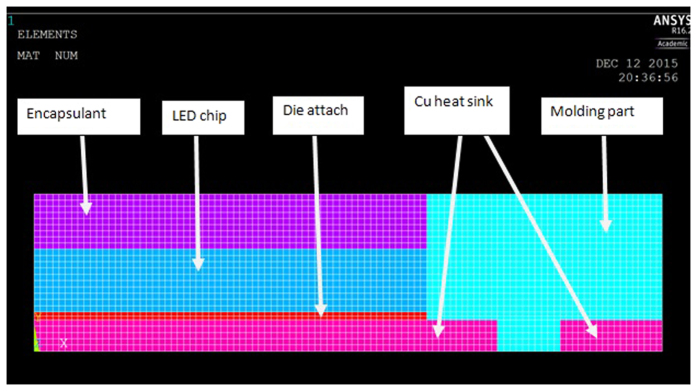
LED structure used for Ansys simulation.

Finite element analysis is employed to provide a quantitative understanding of our proposed mechanisms. ANSYS software is used for the finite element analysis. Figure 9 represents the LED structure used for ANSYS simulation after meshing. One- fourth of LED structure is used for the ease of verification.

For the simulation of LED under power ON condition, the temperature applied on the outer body is 85 °C and the temperature of the LED chip is 135 °C as mentioned earlier. For the simulation of LED under power OFF condition, the temperature on the outer body is the same as that under power ON condition but there is no temperature applied on the inner body of LED. Table 4 shows the parameters of the materials used in the finite element analysis^5^.

**Figure 10:**
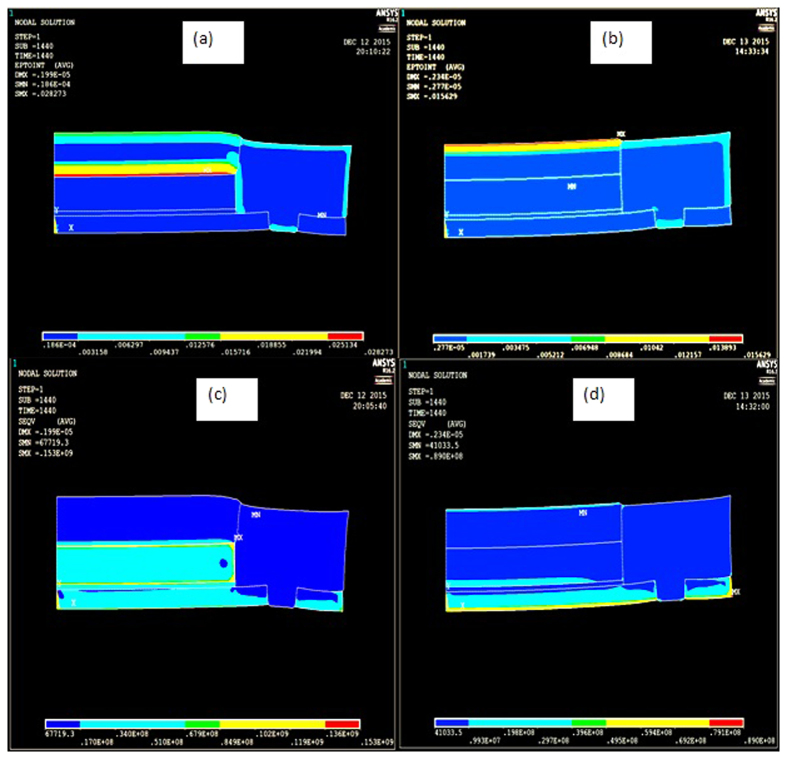
Comparison of mechanical strain in (**a**) LED under On condition and (**b**) LED under Off condition, and von misses stress in (**c**) LED under On condition and (**d**) LED under Off condition.

The distributions of the mechanical strains and von Mises stresses in the LED structure under the above mentioned two conditions as shown in Fig. 10.

It is clearly visible from Fig. 10(a,b) that maximum mechanical strain is at the interface between LED chip and encapsulant for LED under power ON condition, and it is at the outer surface of encapsulant for LED under power OFF condition due to the external temperature. The mechanical strain present at the interface between encapsulant and molding part is also quite significant for LED under both power ON condition. On closer look at their values as summarized in Table 5, we can see that the maximum mechanical strain and von Mises stresses at the interface of chip/encapsulant and encapsulant/molding part for LEDs under power ON condition is significantly larger that those for LEDs under power OFF condition. Also, for LED under power OFF condition, the maximum von misses stress is present at the outer edge of copper heat sink which is very far from the above-mentioned interfaces. Thus, we can conclude that the LEDs under ON condition are more susceptible to the encapsulant delamination from LED chip and molding part, and these delamination lead to a gap that allow moisture to penetrate into the package easily. This results correlate exactly the finding from optical microscope and C-SAM.

The above findings are also consistent with the findings from our electrical measurements as summarized in Fig. 6. I-V measurements are done for the samples to compare the electrical characteristics of the LEDs tested under different conditions. All the degraded samples experience reduction in their forward voltages as shown in Fig. 6. This is explained as follows.

Due to the delamination at the interface of molding part and encapsulation, moisture reaches the die attach of LEDs rapidly under power ON condition. This resulted in the chip-die attach delamination and thus increases their thermal resistance, and hence a reduction in their forward voltages are observed for all the degraded LEDs under power ON condition.

To further quantify the impact of moisture and temperature on the electrical characteristics of LED chips in this MET test, the ideality factor (n), series resistance (Rs) and reverse saturation current (*I*_*s*_) of the pn junction of each tested chip are calculated using the integration based parameters extraction method developed by Tan *et al*.^4^,^7^,^18^. These parameters provide useful information regarding the packaged high power LEDs tested under different conditions^20^,^21^. The results are tabulated in Tables 6 and 7 for white and blue LEDs respectively.

The ideality factor of a pn junction is a measure of the level of carrier injection through the junction^22^. The computed ideality factors for the junction in LEDs are found to be from 3.1 to 4, which are well within the healthy range that has been reported of 2.0–7.0 4. In a PN junction, the reverse saturation current *I*_*s*_ is due to the diffusive flow of minority electrons from the p-side to the n-side and the minority holes from the n-side to the p-side. Hence, the reverse saturation current depends on the diffusion coefficient of electrons and holes as well as the energy barrier at the junction for the carriers. *I*_*s*_ value is very small, and hence any slight change in the chip electrical characteristics will cause a large change in its value, making it the most sensitive indicator for chip degradation. Its value is not measured, but it is determined by a special algorithm as described in ref. 18. This algorithm has been used successfully in many applications as shown in the refs 4, 7, 18 and 19.

When a pn junction is shunted due possibly to ionic contamination or moisture, an electrical path is formed and thus current can flow through this path under reverse bias, increasing the value of *I*_*s*_. Thus, an increase in the value of *I*_*s*_ alone, i.e. the values of n and Rs do not change significantly, represents a formation of shunt path across a pn junction.

As seen from Tables 6 and 7, no order of magnitude change in the values of n, Is and Rs is observed for white LEDs under both the power-ON and OFF conditions, indicating that there is no LED chip related failure in this case. This can be explained as follows.

For the case of white LEDs under power-on condition, since the light absorption by the phosphor layer is also a source of heat generation in LEDs^23^,^24^,^25^, the moisture entered into the package via the delamination at the encapsulation/molding part interface is driven away by the internal heat from the phosphor layer, leaving no moisture reaching the chip, and hence *I*_*s*_ value does not change. This shows that the phosphor layer used to convert blue light into yellow light in white LEDs indeed protects the LED chip from degradation due to moisture in MET test. On the other hand, the higher internal heat from both the chip and the phosphor in the white LEDs causes the LEDs’ package to degrade more severely than the blue LEDs.

On the other hand, this is not the case for the blue LEDs. We can see that *Is* value decreases significantly under power On condition whilst its value increases significantly under power OFF conditions. For example, *I*_*s*_ is reduced by 1000 times for blue LEDs tested under ON condition for 365 hours, while its n and R_s_ values change only slightly. This may be due to the presence of ionic contamination at the junction in the fresh LEDs, and the incoming moisture that reaches the junction dissolves the contamination. However, the high junction temperature of LEDs under the power ON evaporates moisture away from the LED junction, which in turn also removes the contamination dissolved in moisture, thereby decreasing the reverse leakage current of the junction. C-SAM images in Fig. 8 clearly verify that moisture is present at the parameter of the LED chip for blue LED under OFF condition whereas it is absence for the LED chip under ON condition. On the other hand, when blue LEDs are in the power-off condition, moisture reaches the LED perimeter and stays there due to the lack of heat that can evaporate the moisture. Thus, the LED chip is protected from the incoming moisture under power ON condition, but this heat also affects the die attach materials as well as the surrounding packaging materials resulted in higher lumen degradation when compared with blue LED under OFF condition. The reduction of Vf in Fig. 6(b) is a clear indication of the increase in the thermal resistance of LEDs tested under the power ON condition.

### Investigation of lumen recovery stage in LEDs

**Figure 11:**
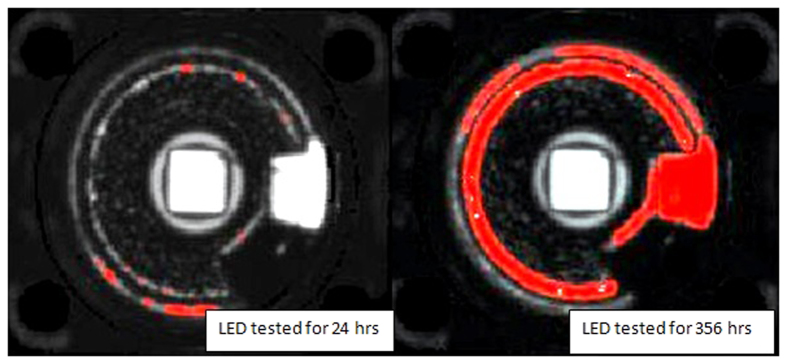
C-SAM images for comparison of Blue LEDs before and after recovery stage. The red areas show where the cracks/delamination occurs when the samples are exposed to high temperature and humidity exposures. The left side image shows the blue LED’s C-SAM image tested for 24 hours and right side image is the degraded blue LED’s C-SAM image tested for 356 hours. The die attach area can be identified easily by a square exactly at the center of the LED package.

To investigate the moisture penetration into the blue LEDs packages under their power-on condition, the LEDs before recovery period, i.e. tested for only 24 hours and after recovery period (tested till the end of test) are investigated using C-SAM. For the initial degradation stage as shown in Fig. 11(a), very little delamination is visible when compared to the LEDs after complete testing in Fig. 11(b). This shows that the initial degradation is due to the moisture trapped in silicone encapsulation as shown by Tan *et al*.^4^ whereas the later percentage lumen degradation is due to the large amount of moisture penetrating via this gap created at the interface between silicone encapsulation and molding part that lead to various steps of degradation as shown by Tan *et al*.^7^.

**Figure 12:**
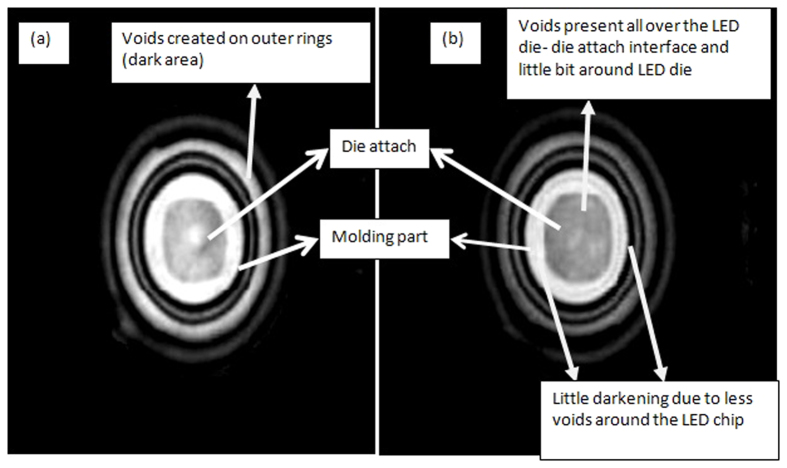
C-SAM of flipped blue LED (**a**) before initial degradation (tested for 24 hours) and (**b**) after final degradation (tested for 356 hours). Die attach interface with LED chip got completely blackened after testing for 356 hours whereas area outside LED chip is not so darkened. The die attach area can be identified easily by a square exactly at the center of the LED package.

To verify the effect of moisture penetration into the LEDs’ packages, C-SAM images for LEDs flipped upside down (i.e. the lens is facing downward) are taken for the blue LEDs under power-on condition before recovery and after the complete test. The small black dots in the C-SAM images are small voids (trapped bubbles) at the molding part/die paddle interface and the LED chip/die attach interface. The die attach area can be identified easily by a square at the center of the LED package in Fig. 12.

The entire surface of the die attach is filled with black dots due to the voids created by moisture for the blue LEDs under power ON condition after the complete test as shown in Fig. 12(a). This shows that the gap created at the interface between molding part and encapsulation allows lot of moisture to penetrate through and reach the die attach and also the top surface of the LED die which led to an increase of percentage lumen degradation. This is in accordance with the results shown in Table 7 for blue LEDs where *I*_*s*_ is decreased 1000 times as explained earlier, however for the LEDs tested only till 24 hours, i.e. before recovery period, only 10 times decrease in the *I*_*s*_ value is observed. This large change in *I*_*s*_ values indicates that the moisture is evaporating away from the LED chip due to high junction temperature as testing prolonged, improving the *I*_*s*_ values. As the high junction temperature also causes die attach to delaminate due to differential thermal expansion between the chip and the die attach material, the von Mises stress is maximum here as shown in Fig. 10, the die attach is sucking up most of the moisture entering from the gap created in between molding part and encapsulant as it is the only path available for the moisture to penetrate, and this reduces the amount of moisture present in the encapsulant. Thus, the light from the LED chip is less scattered by the moisture in the encapsulant, and lumen degradation recovery is observed after the initial lumen degradation in first 24 hours.

### Comparison of degradation of LEDs under MET test and other tests with single or double stresses

Reliability studies on high power LEDs have been extensive, but most of them are done with either single stress or double stress effect on LEDs. Generally, single stress is either electrical stress or operating temperature stress, and double stresses could be the combination of electrical at high temperature or high temperature-humidity tests. The failure modes and failure mechanisms involved with these single, double and triple stresses on the LEDs are summarized in Table 8.

From Table 8, we can see the differences in failure mechanisms for LEDs when tested under different stresses. For example, LEDs experienced rapid light output degradation due to browning of the white silicone reflector molding part of device and encapsulant detachment from molding part which created a wide and easy path for moisture to penetrated inside the LED package and create further damage to LED under MET test. This failure mechanism is unique to MET test, and it shows that different reliability test should be performed for the evaluation of reliability of LEDs under different operating conditions, depending on their applications. For example, for indoor usage of LEDs, electrical test alone may be sufficient if good heat sink is incorporated; if heat sink is poor, then electrical-thermal test will be suitable. However, for outdoor usage, MET is needed, and temperature-humidity may also be needed during the period when LEDs are not turned on, such as in the morning. In applications like marine and automobiles, LEDs will face all the three stress factors i.e. high temperature, moisture and electrical stress at the same time when LEDs are turned on, and MET test will be needed. MET test could help the LED industry to understand the new failure mechanisms for LEDs when used in outdoor or harsh conditions.

## Conclusion

A new MET test is proposed and performed for LEDs where the combined effect of moisture, electric and temperature stress is observed with and without the presence of phosphor. White LEDs (i.e. with the presence of phosphor) show rapid percentage lumen degradation as compared to blue LEDs (without phosphor) when tested under MET test. The gap created at the interface of the molding part and encapsulation material due to difference in coefficient of thermo-expansivities among the two materials is found to be the main degradation reason in this test. It allowed a large passage of moisture to enter the LED package and cause severe degradation. The presence of moisture is found to be responsible for higher lumen degradation as well as the reduction of forward voltage in I-V characteristics for LEDs under ON condition when compared with LEDs under power OFF condition for both type of LEDs. The presence of phosphor in white LEDs provides protection for LED chip from moisture as the heat accumulation in white LEDs phosphor evaporates the moisture from the LED die surface. However, this phosphor also causes browning of the white silicone reflector molding part of device which led to encapsulant detachment from molding part that in turn led to 30% lumen degradation in just 144 hours of testing. Thus, white LEDs using phosphor is not as reliable as expected as observed in this work. RGB LEDs without phosphor integrating 3 colors red, green and blue to produce white light could be a better option than white LEDs having blue or UV LEDs with phosphor integrated into it for future solid state lighting. However, no reliability study on such RGB white LED is reported.

The necessity of the MET test is also demonstrated by comparing the failure mechanisms occur in this test with other single or double stress factors. In fact, different reliability tests should be performed for the evaluation of LED reliability depending on its application as the corresponding failure mechanisms can be very different.

## Additional Information

**How to cite this article**: Singh, P. and Tan, C. M. Degradation Physics of High Power LEDs in Outdoor Environment and the Role of Phosphor in the degradation process. *Sci. Rep.* **6**, 24052; doi: 10.1038/srep24052 (2016).

## Figures and Tables

**Table 1:** Test conditions of LEDs for Experimentation.

Test condition	White LEDs		Blue LEDs	
	Sub-set A1 (20 samples)	Sub-set B1 (20 samples)	Sub-set A2 (20 samples)	Sub-set B2 (20 samples)
Power condition	On (with 350 mA)	Off	On (with 350 mA)	Off
Temperature/humidity conditions	85%RH/85 °C	85%RH/85 °C	85%RH/85 °C	85%RH/85 °C

**Table 2:**
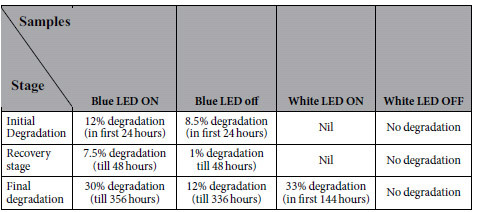
Time taken for initial degradation, recovery and final degradation by blue and white LEDs under different operating conditions.

**Table 3:** Conditions and Rationale of the 6 chosen samples for I-V examination and C-SAM spectroscopy.

Unit #	Test condition undergone by the test unit	White/Blue LEDs	Rationale of the choice	Remarks
1	Power on-condition, tested for 144 hours	White	Maximum degraded sample with 40% lumen depreciation	Tested stopped after 144 hours as LEDs under On condition reached 70% of its initial lumen output, following the ASISST standards^13^
2	Power off-condition, tested for 144 hours	White	Maximum degraded sample with 2% lumen depreciation	
3	Power on-condition, tested for 356 hours	Blue	Maximum degraded sample with 32% lumen depreciation	Tested stopped after 356 hours as LEDs under On condition reached 70% of its initial lumen output, following the ASISST standards^13^
4	Power off-condition, tested for 356 hours	Blue	Maximum degraded sample with 13% lumen depreciation	
5	Power on-condition, tested for 24 hours	Blue	Maximum degraded sample with 13% lumen depreciation	Samples are chosen from the respective sets to observe the effect of MET test before and after “degradation recovery”.

**Table 4:** Thermal and mechanical properties of the materials used in the finite element analysis.

Material	Density, ρ (kg/m^3^)	Specific heat, C (J/(kg.K))	Thermal conductivity, K (W/m.K)	Elastic modulus, E (GPa)	Poisson ratio	Coefficient of thermal expansion, α, CTE (e-6/°C)
Molding part	1100	1591	0.209	2.3	0.394	80
Encapsulant	1200	1260	0.2	1	0.4	220
Die attach	7390	217	57	52.6	0.35	20
LED chip	3965	730	130	210	0.17	7.75
Copper heat sink	8950	390	400	110	0.34	18

**Table 5:** Comparison of mechanical strain and von misses stress at the LED chip- encapsulant interface and encapsulant- molding part interface for LEDs under On and Off conditions.

	LED chip – Encapsulant interface		Encapsulant – Molding part interface	
	LED On state	LED Off state	LED On state	LED Off state
Maximum Mechanical strain	0.0287	0.27E − 5	0.009	0.27E − 5
Maximum Von- Misses stress (Pascals)	0.15E + 9	4.1E + 4	6.7E + 4	4.1E + 4

**Table 6:** Summary of the n, Rs and Is values for the tested packaged white LEDs.

	Fresh White LED	White LED under power-off condition after 144 hours	White LED under power-on condition after 144 hours
N	2.39	2.35	2.32
Is (A)	2.35 × 10^−22^	1.7 × 10^−22^	2.08 × 10^−22^
Rs (Ω)	1.16	0.97	0.84

**Table 7:** Summary of the n, Rs and Is values for the tested packaged blue LEDs.

	Blue LED fresh	Blue LED under power-on condition after 356 hours	Blue LED under power-off condition after 356 hours	Blue LED under power-on condition before recovery(tested for only 24 hours)	Blue LED under power-off condition before recovery (tested for only 24 hours)
n	2.44	2.09	3.83	2.33	2.81
Is (A)	1.83 × 10^−20^	7.43 × 10^−23^	7.95 × 10^−14^	5.95 × 10^−21^	5.03 × 10^−18^
Rs (Ω)	0.96	1.00	0.87	1.13	0.92

**Table 8:** Summary of The failure modes and failure mechanisms involved with these single, double and triple stresses on the LEDs.

External stress(es) applied	Failure sites	Failure modes	Failure mechanisms	References
Electrical stress	Chip Level	Lumen degradation, increase in reverse leakage current, increase in parasitic series resistance and short circuit.	Defects in LED chip, electromigration of the metal atoms in the electrical contact to the surface of the LED die, instability in Mg diffusion in p- GaN layer and dislocation generation and movement at chip level	26,27,28,29,30,31,32,33,34,35,36
	Package level	Lumen degradation	Carbonization of encapsulation and phosphor thermal quenching	3,4,5,6,7,8,9,10,11,12,13,14,15,16,17,18,19,20,21,22,23,24,25,26,27,28,29,30,31,32,33,34,35,36,37,38,39,40,41,42,43
Thermal stress	Package level	Lumen degradation, Detachment of encapsulant from molding part	Browning of the white reflector molding part of device	44
	Chip level	Lumen degradation	Degradation of the phosphors conversion efficiency	20, 44, 45
Electrical stress and thermal stress	Chip level	Lumen depreciation	Crack in the LED chip	30, 31
	Package level	Lumen degradation	Encapsulant yellowing, lens cracking or solder joint fatigue	46, 47, 48, 49, 50
Thermal stress and moisture stress	Chip level	Lumen degradation, Forward voltage change, permanent destruction of LED i.e. no light	Wire ball bond fatigue	7
	Package level	Reduction of the overall light output,	LED chip-die attach delamination, moisture entrapment in encapsulant, and lens cracking	4, 5, 6, 7, 8, 51, 52
Thermal, electrical and moisture stresses (MET test proposed in this work)	Chip level	Rapid Lumen depreciation, forward voltage drop	LED chip/die attach delamination, defects in LED chip	Proposed mechanism in this paper
	Package level	Rapid Lumen degradation	Browning of the white silicone reflector molding part of device, encapsulant detachment from molding part	Proposed mechanism in this paper
